# Perceived Deterrence Towards Colonoscopy for Colorectal Cancer Screening among Northern Malaysia Population: A Qualitative Study

**DOI:** 10.31557/APJCP.2020.21.5.1253

**Published:** 2020-05

**Authors:** Mohd Azri Mohd Suan, Wei Leong Tan, Ibtisam Ismail, Muhammad Radzi Abu Hassan

**Affiliations:** 1 *Clinical Research Center, Hospital Sultanah Bahiyah, Alor Setar, Kedah, Malaysia. *; 2 *Kedah State Health Department, Alor etar, Kedah, Malaysia. *; 3 *Medical Department, Hospital Sultanah Bahiyah, Alor Setar, Kedah, Malaysia. *

**Keywords:** Colonoscopy, colorectal cancer, deterrence, screening, Malaysia

## Abstract

**Background:**

Patients with positive immunochemical faecal occult blood test results were found to have poor compliance for a subsequent colonoscopy procedure. This study was conducted to explore patients’ perceived deterrence for colonoscopy following a positive stool test.

**Methods:**

Using qualitative study method, a phone interview was conducted with 16 patients to elicit their views on the reasons for failure to attend the colonoscopy procedure following a positive stool test. The interviews were audio recorded, transcribed verbatim and translated before proceeded with the data analysis. Content analysis was made on the translated interview, followed by systematic classification of data by major themes.

**Results:**

Reasons for nonattendance were categorized under five main themes; unnecessary test, fear of the procedure, logistic obstacles (subthemes; time constraint, transportation problem), social influences, and having other health priority. Lacking in information about the procedure during the referral process was identified to cause misperception and unnecessary worry towards colonoscopy. Fear of the procedure was commonly cited by female respondents while logistic issues pertaining to time constraint were raised by working respondents.

**Conclusions:**

More effective communication between patients and health care providers are warranted to avoid misconception regarding colonoscopy procedure. Support from primary care doctors, customer-friendly appointment system, use of educational aids and better involvement from family members were among the strategies to increase colonoscopy compliance.

## Introduction

Colonoscopy is stated in many international guidelines (European Colorectal Cancer Screening Guidelines Working Group et al., 2013; Sung et al., 2015) as the gold standard among other imaging modalities to detect colorectal cancer, the third most common cancer in the world (World Cancer Research Fund International, 2017). Colonoscopy is not only served as a screening tool but also as a therapeutic tool to remove any precancerous adenomas at an earlier and treatable stage (Zauber et al., 2012). In the hand of a trained operator, colonoscopy detection rates of adenoma and colorectal cancer for individuals above 50 years old were between 7.4 to 54.2% (Corley et al., 2014) and around 0.1-1.0% (Sung et al., 2015), respectively. 

In addition to the quality of examination, the effectiveness of colonoscopy depends on the screening uptake. In a trial involving eight Spanish regions, the participation rate for colonoscopy was lower than for iFOBT (24.6% vs 34.2%), which hampered the advantages of colonoscopy in detecting and removing precancerous lesion (Quintero et al., 2012). Adherence to colonoscopy following a positive iFOBT was also suboptimal. According to the report from Lithuania National Colorectal Cancer Screening Program, only 52.4% of the iFOBT-positive individuals undergo colonoscopy, resulting in a meagre colorectal cancer detection rate (Poskus et al., 2015). Other reported factors that discourage patient from undergoing colonoscopy were comorbid conditions, competing life priorities, transportation difficulties and lack of social support (Jetelina et al., 2019). 

Despite a high national incidence and mortality rate from colorectal cancer (Abu Hassan et al., 2014), colorectal cancer screening in Malaysia is still at its infancy stage. Predominantly, colorectal cancer screening available in Malaysia is iFOBT complemented by colonoscopy (Abu Hassan et al., 2016). A local study in Northern Malaysia showed that the screening uptake of iFOBT was relatively good (94.7% for first FOBT and 90.7% for second FOBT), nevertheless, colonoscopy compliance was suboptimal among patients with positive iFOBT (Abu Hassan et al., 2016). Various deterrence may have hindered local patients’ compliance for a colonoscopy; however, it was not further explored. Thus, we conducted this study to explore deterrence for colonoscopy following positive iFOBT from the patients’ perspective.

## Materials and Methods

*Study design and setting*

A qualitative study was conducted using an in-depth interview via telephone among patients with positive iFOBT who refused colonoscopy. The study participant was selected among patients that joined a colorectal cancer screening program conducted at all government district health clinics in Kedah state between April and December 2016. In brief, this screening program targeted patients of 50 years of age or older, have no symptom of colorectal malignancy, and have no family history of colorectal cancer. Patient who fulfilled these criteria were invited to participate in the colorectal cancer screening program. Patients who agreed to join the program were given the iFOBT kit and guide on stool self-collection. Stool analysis was conducted after the patients sent their samples to the health clinics. Patients with a positive iFOBT result were referred to a nearby hospital with endoscopy facilities for colonoscopy. 

*Participants*

We used purposive sampling and potential respondents were identified based on the list of patients with a positive iFOBT result and referred to the hospital for colonoscopy, retrieved from the participating clinics. The list contained patient demography information such as age, gender, ethnicity, phone number and residential status. Phone calls were then made to all patients in the list between January and April 2017 to ascertain whether they had undergone the colonoscopy procedure. Participants who defaulted for colonoscopy despite being referred for the procedure were interviewed further. 

*Interview*

The interview aimed to explore the participants’ perceived deterrence towards going through a colonoscopy procedure. A semi-structured interview guideline was developed based on literature reviews and input from the gastroenterologist to ensure key topics related to the study objectives were covered in each interview. All participants were informed regarding the objective of the study at the beginning of the interview. Two of the authors (MAMS and WLT), male medical doctors with experience in clinical and public health research, conducted the interviews over the phone with eligible participants in a private room at Clinical Research Center. Both interviewers also have prior experiences with qualitative interviews before conducting this study. Another female author (II) assisted in each interview session as note-taker. All authors did not know the participants prior to interview session and introduced themselves to the participants before commencing the interview. The interviews lasted approximately 30 to 45 minutes using the national language (Bahasa Malaysia). The recruitment of new participants for interview continued until the data saturation was reached. All interviews were audio recorded, transcribed verbatim and translated before the thematic analysis. All participants provided informed verbal consent prior to the interview.

*Data Analysis*

Data analysis was conducted using the thematic analysis method. Together with the note taken, the data-rich interview texts were transcribed, translated, read and reread by three authors to familiarise themselves with participants’ responses. The authors collated similar features in the data to generate codes. All codes were then re-analysed and combined to form themes and sub-themes. The interpretation of the themes was further validated by discussions between the authors and the gastroenterologist. The final themes were then used in the study report. The study methods and findings were reported according to the consolidated criteria for reporting qualitative research (COREQ) (Tong et al., 2007).

*Ethical approval*

This study has received the approval from the Medical Research and Ethics Committee, Ministry of Health Malaysia (NMRR-16-397-29665).

## Results

*Participants’ characteristics*

From the 118 patients who did the iFOBT test between April and December 2016, 41 patients have been identified as having a positive iFOBT and were then referred for colonoscopy. However, there were only 23 patients underwent the colonoscopy procedure when contacted by the investigators/authors. Two patients were unable to be reached for the interview and the rest (n=16) were giving various reasons for noncompliance to colonoscopy referral. The studied participants were mostly from Malay ethnicity and of female gender. Other characteristics of participants interviewed were summarised in Table 1. Five themes (unnecessary test, fear of the procedure, logistic obstacles [subthemes: time constraint and transportation problem], social influences, and had other health priority) emerged from the interview data relating to the participants’ perceived deterrence towards colonoscopy following a positive iFOBT screening.

*Misperception about colonoscopy*

Eleven out of 16 participants (69%) perceived colonoscopy as an unnecessary procedure when asked about their reasons for declining colonoscopy. The most common excuse for the test being viewed as unnecessary was that they were feeling healthy. In particular, the participants claimed that they had neither abnormal bowel symptoms nor abdominal pain. 

*“I don’t have any (bowel) problem. No pain, nothing. I don’t think that I need to do that procedure (colonoscopy).” – Participant #2.*

*“I have done the stool test (iFOBT) while I’m healthy. Now they (the health clinic staff) asked me to go for colonoscopy for no reason. This is (the referral for colonoscopy) unnecessary!” – Participant #11.*

Some respondents also saw the colonoscopy procedure as unnecessary because they had no family history of colorectal cancer. 

*“I was told that those with a family history of colon cancer that should go for colonoscopy. So why me? None of my family members had it (colorectal cancer).” – Participant #16.*

*Fear of the procedure*

Many of the noncompliant participants did express their anxiety towards the procedure, such as fear of the procedure, thought about embarrassment, and fear of pain. 

*“I don’t want to go for colonoscopy. I’m worried that they will put a tube through my nose during the procedure. I have experienced that (tube being put in the nose) in my previous admission to the ward.” – Participant #1.*

*“Who is going to be there (during the procedure)? Are they (the staff performing the procedure) going to take off all of my clothes? …. I don’t feel comfortable to go for it (colonoscopy).” – Participant #8*

*“I have heard from my neighbours about how painful the colonoscopy is. I could not sleep at night thinking of the procedure.” – Participant #14*

Further to questioning these participants, most of them admitted that not much information was given to them during the referral process.

*“I was called to the clinic. Then I received the referral letter for colonoscopy and advised to adhere to the appointment date. Not much was said on the procedure itself.” – Participant #14.*

*Logistic obstacles*

Several logistic problems were also reported by several participants as deterrence for not attending the colonoscopy appointment. These logistic issues were categorised into two subthemes. 

*Time constraint*

Being busy was the most common reason for not attending colonoscopy given by the participants who were still working. Although they were informed that the colonoscopy appointment could be rescheduled to accommodate the patients’ time, the participants did not feel that it (rescheduling the appointment) should be done sooner. 

*“I could not arrange time off from my work to attend the (colonoscopy) appointment...” – Participant #15.*

*“Not yet. I’m tight with my work here… (But sir, you can call the clinic or hospital and get a new appointment that may suit to your time). Oh really? Maybe later. I have lots of other important things to do now.” – Participant #13.*

*Transportation problem*

As many of the participants were in the old age group, they were dependent on their adult children to drive and accompany them to the hospital for the appointment. 

*“I’m too old to drive myself to the hospital. I need to wait for my son to bring me to the hospital (for colonoscopy appointment).” – Participant #3.*

*“I have called and informed my daughter about it (colonoscopy). She said to wait for her. She will come next month and accompany me to see the doctor.” – Participant #5.*

*Social influences*

Influences and opinions from other people would affect the respondents’ decision about the colonoscopy procedure. The comments can be found in the following quotes.

*“When I informed my children about the stool test result and the need to go for colonoscopy, they did not agree with it (going for colonoscopy).” – Participant #4*

*“My friends told me that they already had colonoscopy three times! And already had an appointment for the fourth. Why do you need to insert that camera so many times? I don’t feel like starting one.” – Participant #5*

*Had other health priority*

Some of the participants have put their existing health problems as a priority to be treated first before willing to go for colonoscopy. Examples of responses varied from ‘waiting for blood test results’ to a more chronic health condition.

*“I had this problem with my bladder. I could not control my urine. It was getting worse lately. My doctor already took some blood and urine sample for a test. The result will come out soon and if normal (the blood/urine test), I will go for the colonoscopy.” – Participant #9.*

*“I just had a mild stroke three months ago. I guess the colonoscopy need to wait until I recover from the stroke.” – Participant #12.*

**Table 1:** Participants Characteristics

ID	Age	Gender	Ethnicity	Residential status
#1	52	Female	Malay	Nonurban
#2	56	Male	Malay	Nonurban
#3	79	Male	Malay	Urban
#4	60	Female	Malay	Nonurban
#5	66	Male	Indian	Nonurban
#6	75	Male	Malay	Nonurban
#7	68	Female	Malay	Urban
#8	54	Female	Chinese	Nonurban
#9	60	Female	Malay	Urban
#10	51	Female	Malay	Urban
#11	59	Female	Malay	Urban
#12	55	Male	Malay	Urban
#13	54	Female	Malay	Nonurban
#14	63	Female	Malay	Urban
#15	51	Male	Chinese	Urban
#16	56	Female	Chinese	Urban

## Discussion

As of known, this is among the first local study using a qualitative method to explore the deterrence for colorectal cancer screening test, particularly on colonoscopy. The results highlight several important issues towards adhering to colonoscopy among patients with a positive iFOBT result. There were almost half of iFOBT-positive respondents in the studied population that did not attend the colonoscopy appointment. Therefore, this study provides a good insight into the underlying reasons for colonoscopy incompliance from the patients’ perspective. 

Various reasons were given by the participating patients with a majority of them perceived colonoscopy as an unnecessary test. Based on their responses, the patients believed that having symptoms or positive family history is necessary for colorectal screening. Earlier study by Wools et al., (2016) suggested that feeling of susceptibility to the disease played a major role in patients’ participation in the colonoscopy. Patients feeling healthy and not vulnerable to colorectal cancer leads to lower participation in the screening test, which was shown in the current study. On top of that, patients without a family history of colorectal cancer have a lower tendency in undergoing colonoscopy (Prunuske et al., 2010).

The perception of low susceptibility to cancer could be related to the patients’ knowledge of colorectal cancer and its screening test (Carnahan et al., 2019). Although the present study did not assess the patient’s knowledge of colorectal cancer or colonoscopy, findings from other local studies shown that low patients’ knowledge correlates with poor screening uptake (Koo et al., 2012; Lim, 2014). Knowledge on the risk of colorectal cancer is significantly associated with the decision of colonoscopy acceptance even in a developed country (Adler et al., 2014). Hence, better health promotion needs to be done to enlighten the public regarding the devastating impacts of colorectal cancer and the importance of its screening test. 

Many patients also referred to thoughts of fear or embarrassment as deterrents to completing colonoscopy procedure. This finding is in line with other quantitative study outcomes (Adler et al., 2014; Lee, 2018; Shelton et al, 2011). For instance, a study in Berlin, Germany found that most reason given for not having colonoscopy among the studied population were related to fear, discomfort or concern about the colonoscopy procedure (Adler et al., 2014). Likewise, a study conducted in Republic of Korea indicated that pain, discomfort and embarrassment were the perceived barriers for the patient to adhere to colorectal cancer screening tests (Lee, 2018). The current study also found that such comments mainly cited by female respondents, which could be due to their concern on modesty during the procedure. 

Furthermore, several patients also commented on the referral process as lacking in information about the procedure, which may subsequently expose patients to the unnecessary worry and fear that could lead them not turning up for the colonoscopy. The finding that local patients fail to prioritise their health concern may also have resulted from inadequate information conveyed during referral. The authors believed that the patient and health clinic staff should have a more informative discussion during the referral process, not only to avoid the misperception on colonoscopy but also to guide those with multiple comorbidity to set their health priority without ignoring the screening test. 

Thus, a dedicated team at the health clinic is indeed needed to counsel these iFOBT-positive patients before colonoscopy referral. It is also imperative to engage the primary care doctors and family physician in the counselling team to convey the essential knowledge of colorectal cancer and colonoscopy. Any patients’ queries regarding the procedure can be resolved during the counselling to increase their colonoscopy compliance. The role of family physicians in recommending patients for colorectal cancer screening has been proven effective in previous studies (Hewitson et al., 2011; Stanley et al., 2019). Also, the use of several educational aids, such as printed material (e.g. pamphlet) or audio-visual (e.g. short video), can help patients in better understanding about colorectal cancer and colonoscopy procedure. It is also suggested that such educational materials should include the contact number of the health professional if any queries regarding colonoscopy arise after the counselling session. 

Logistic obstacles to attending the colonoscopy appointment also emerged as an important deterrence in this study. This theme was divided into two subthemes; time constraint and transport problem. In addition to colonoscopy, time constraint was also causing low adherence to other screening programs/tests (Brown et al., 2013; Mohd Suan, 2015; Waller et al., 2012). A tight schedule is more commonly observed among working patients. Some studies have recommended several alternative appointment methods (e.g. test offered over the weekend, late evening appointment, having different referral routes for diagnostic and screening test) to ease patients to attend the screening test (Inadomi et al., 2019; Ryan et al., 2019). The authors believed that the appointment system needs to be customer-friendly (i.e. phone-based appointment booking, avoiding repeated appointment visits for the colonoscopy procedure, etc.) to avoid distress and noncompliance to the patients. 

Several studies also indicate that family and friends’ opinions and experiences had a great influence on the patient’s decision to participate in the colorectal screening test (Lynsey et al, 2019). Moreover, there are elderly patients in the local population who were dependable on their adult children for companion and transportation to visit the hospital (Alavi, 2013). As a result, this scenario prevented some patients from completing the colonoscopy as the adult children have another commitment. To overcome this problem, family members need to be involved during the counselling session. Perhaps, by attending such session, the family members can view the importance of completing the screening procedure and provide necessary assistance (accompanying, transport) for the patient. 

Some limitations need to be noted here. First, our data analysis on deterrence for attending colonoscopy procedure consisted of a small purposive sampling respondent from a single state, which limits the findings’ generalization. Nonetheless, this study was warranted as a lack of local research being done using qualitative method to address the issue of poor colonoscopy uptake. The authors also recommend a future study to be conducted on population with different ethnic composition to get a better understanding of the deterrence towards colonoscopy noncompliance. Second, the perceived deterrence for colonoscopy were based on patients’ perspectives. Other health personnel’s (endoscopy operator, gastroenterologist, health manager) views should also be considered if the plan to reduce any deterrence impeding patients from going through the colonoscopy procedure. Third, this study only focused on deterrence towards colonoscopy, while other screening tools to detect colorectal cancer were not discussed. As the state screening program for colorectal cancer just started a few years ago, a detailed list of patients that underwent iFOBT was well kept and prompted the researcher to concentrate on the issues surrounding colonoscopy referral. Other screening modalities for colorectal cancer detection may have different reasons for poor compliance and we hope to explore in the coming study. 

In conclusion, the present study provides valuable knowledge of various deterrence that influence the low turn-up for colonoscopy referral in the studied population. Inadequate information was the main factor that triggers most deterrence for patients from attending the colonoscopy procedure. Thus, continuous effort is needed from health care providers to educate and disseminate essential information regarding colonoscopy to the local public. Other strategies to improve the screening test uptake such as family involvement, attentive educational aids, and alternative appointment methods, were equally important for health betterment of the society. 
